# Bark Extracts of Ceylon Cinnamon Possess Antilipidemic Activities and Bind Bile Acids In Vitro

**DOI:** 10.1155/2017/7347219

**Published:** 2017-07-20

**Authors:** Walimuni Prabhashini Kaushalya Mendis Abeysekera, Sirimal Premakumara Galbada Arachchige, Wanigasekera Daya Ratnasooriya

**Affiliations:** ^1^Herbal Technology Section (HTS), Modern Research & Development Complex (MRDC), Industrial Technology Institute (ITI), 503A Halbarawa Gardens, Malabe, Sri Lanka; ^2^Department of Zoology, Faculty of Science, University of Colombo, Colombo, Sri Lanka; ^3^Faculty of Allied Health Sciences, General Sir John Kotelawala Defence University, Ratmalana, Sri Lanka

## Abstract

Ethanol (95%) and dichloromethane : methanol (1 : 1) bark extracts of authenticated Ceylon cinnamon were investigated for range of antilipidemic activities (ALA): HMG-CoA reductase, lipase, cholesterol esterase, and cholesterol micellization inhibitory activities and bile acids binding in vitro. Individual compounds in bark extracts were also evaluated. Bark extracts showed ALA in all the assays studied. The IC_50_ (*μ*g/mL) values ranged within 153.07 ± 8.38–277.13 ± 32.18, 297.57 ± 11.78–301.09 ± 4.05, 30.61 ± 0.79–34.05 ± 0.41, and 231.96 ± 9.22–478.89 ± 9.27, respectively, for HMG-CoA reductase, lipase, cholesterol esterase, and cholesterol micellization inhibitory activities. The bile acids binding (3 mg/mL) for taurocholate, glycodeoxycholate, and chenodeoxycholate ranged within 19.74 ± 0.31–20.22 ± 0.31, 21.97 ± 2.21–26.97 ± 1.61, and 16.11 ± 1.42–19.11 ± 1.52%, respectively. The observed ALA were moderate compared to the reference drugs studied. Individual compounds in bark extracts ranged within 2.14 ± 0.28–101.91 ± 3.61 and 0.42 ± 0.03–49.12 ± 1.89 mg/g of extract. Cinnamaldehyde and gallic acid were the highest and the lowest among the tested compounds. The ethanol extract had highest quantity of individual compounds and ALA investigated. Properties observed indicate usefulness of Ceylon cinnamon bark in managing hyperlipidemia and obesity worldwide. Further, this study provides scientific evidence for the traditional claim that Ceylon cinnamon has antilipidemic activities.

## 1. Introduction

Hyperlipidemia, a disorder of lipid metabolism, is characterized by the elevated levels of serum lipids [1]. It is considered as one of the five leading causes of death in the world [2]. Several factors, such as diet high in saturated fats and cholesterol, lack of proper physical activity, stress, and other lifestyle factors, greatly influenced the prevalence of hyperlipidemia worldwide [1]. It also plays a central role in pathogenesis of certain noncommunicable diseases such as diabetes, obesity, hypertension, and cardiovascular diseases [3].

Treatment of hyperlipidemia involves diet control, exercise, use of lipid-lowering diets, and drugs [4]. Currently, there are six categories of antilipidemic drugs available in the market, namely, HMG-CoA reductase inhibitors (statins), for example, lovastatin; bile acid sequestrants (anion-exchange resins), for example, cholestyramine and colestipol; fabric acid derivatives (fibrates), for example, clofibrate, gemfibrozil, fenofibrate, ciprofibrate, and bezafibrate; nicotinic acid, for example, niacin; cholesterol absorption inhibitors, for example, ezetimibe; and omega-3-fatty acids (fish oil), for example, Pulse [5]. Although, these allopathic antihyperlipidemic drugs are highly effective, their popularity has been marred by numerous side effects such as myalgia, arthralgia, elevated liver enzymes, elevated blood glucose, dyspepsia and constipation, severe contraindications, drug interactions, and exuberant cost [6]. Obviously, this has led to the search of novel lead molecules preferably from natural products possessing antilipidemic properties with lesser or no side effects and that are less expensive compared to the available antilipidemic drugs. For instance, there are ample evidences elucidating antilipidemic activity of natural products via multiple mechanisms [5, 7–9].

Cinnamon, one of the oldest and most frequently consumed spices worldwide, belongs to the genus* Cinnamomum* and there are different species of cinnamon worldwide [10]. However, the genus contains only four economically important cinnamon species such as* Cinnamomum zeylanicum* or* Cinnamomum verum *(Ceylon cinnamon or true cinnamon),* Cinnamomum aromaticum* (*Cinnamomum cassia* or Chinese cinnamon),* Cinnamomum burmannii* (Korintje, Java, or Indonesian cinnamon), and* Cinnamomum loureiroi* (Vietnamese or Saigon cinnamon) [11]. Among four species, Ceylon cinnamon is the “true cinnamon” (*Cinnamomum zeylanicum* Blume) the world over based on its unique taste, aroma, and phytochemical composition and it is indigenous to Sri Lanka [10]. Currently, Sri Lanka is the topmost and the only continuous supplier of high quality true cinnamon with 85% of world market share and 14.5% market share for all types of cinnamon worldwide. According to the recent statistics nearly 50% of export earnings of minor agricultural crops in Sri Lanka come from Ceylon cinnamon [12].

Cinnamon is generally recognized as safe when used in therapeutic doses. According to the United States Food and Drug Administration, the amount of cinnamon in commonly found foods are generally safe and well tolerated [13]. Several preclinical in vivo studies also have not shown any significant toxic effects of cinnamon [10].

Sri Lankan traditional medical system documents Ceylon cinnamon as a remedy for number of aliments. The bark of this tree is used to treat dyspepsia, latuleace, diarrhea, dysentery, vomiting, bronchitis, gangrene of the lungs, phthisis, cramps of the stomach, toothache, and paralysis of the tongue and used in massive doses in the treatment of cancer. The steamed bark is used externally as a fomentation on boils and abscesses to prevent suppuration. The oil is useful in application for acute and chronic rheumatism [14, 15]. Further, according to some Sri Lankan traditional physicians, Ceylon cinnamon is too claimed to possess antilipidemic effects by inhibition of lipid synthesis, digestion, and/or absorption. Moreover, cinnamon is reported to have antilipidemic activity in several in vitro [16] and in vivo models [10, 17–19] worldwide. However, very few scientific reports [10, 18, 19] are available on antilipidemic activity of Ceylon cinnamon to date. Further, the studies conducted worldwide so far on antilipidemic activity of authenticated Ceylon cinnamon did not address its effect on HMG-CoA reductase, cholesterol esterase, and cholesterol micellization inhibitory activities and bile acid binding. In this connection, this study was initiated to investigate the antilipidemic potential of bark of authenticated Ceylon cinnamon via HMG-CoA reductase, lipase, cholesterol esterase, and cholesterol micellization inhibitory activities and binding of bile acids via widely used, well established, sensitive, specific, reliable, and reproducible in vitro bioassays [10, 20–22].

## 2. Materials and Methods

### 2.1. Chemicals and Reagents

Porcine pancreatic lipase (PPL, type II), 4-nitrophenyl butyrate (p-NPB), porcine pancreatic cholesterol esterase, oleic acid, phosphatidylcholine, cholesterol, sodium taurocholate hydrate, sodium chenodeoxycholate, sodium glycodeoxycholate, orlistat, epigallocatechin gallate (EGCG), cholestyramine resin, cinnamyl acetate, eugenol, kaempferol, trans-cinnamaldehyde, trans-cinnamic acid, phlorizidin, epicatechin, catechin, 4-hydroxybenzoic acid, gallic acid, and HMG-CoA reductase assay kits (CS 1090) were purchased from Sigma-Aldrich Co., St. Louis, MO, USA. Total cholesterol test kits (BXC0261) were purchased from Fortress Diagnostics, UK, and total bile acid kits (BQ 042A-EALD) were purchased from Bio-Quant Co. (San Diego, CA, USA). All the other chemical reagents used in this study were of analytical grade.

### 2.2. Collection of Alba Grade Cinnamon Bark Samples

Alba grade cinnamon bark samples (alba grade cinnamon has the lowest quill thickness, maximum 6 mm, according to the grading of cinnamon quills based on the quill thickness) [23] were collected from cinnamon factories of L.B. Spices (Pvt), Ltd., Aluthwala, Galle, Sri Lanka, and G. P. De Silva and Sons Spices (Pvt), Ltd., Ambalangoda, Sri Lanka. The samples were authenticated by Dr. Chandima Wijesiriwardena, Principle Research Scientist, Industrial Technology Institute, Sri Lanka, and voucher specimens and photographic evidence are deposited at the Pharmacognosy Laboratory, Herbal Technology Section, Industrial Technology Institute, Sri Lanka. Bark samples were ground, powdered, and stored at −20°C until used for the extraction.

### 2.3. Preparation of Bark Extracts

#### 2.3.1. Preparation of Ethanol Bark Extract

Powdered bark (20 g) was extracted in 200 mL of 95% ethanol for 4-5 h in a Soxhlet extractor (4–6 cycles) until the solvent in the siphon tube become colourless. The extract was filtered and evaporated to dryness under vacuum in a rotary evaporator and freeze-dried (Christ-Alpha 1-4 Freeze Dryer, Biotech International, Germany). Freeze-dried extract was stored at −20°C until used for the analysis.

#### 2.3.2. Preparation of Dichloromethane : Methanol (DCM : M) Bark Extract

Powdered bark (20 g) was extracted into 200 mL of dichloromethane : methanol (DCM : M) at a ratio of (1 : 1 v/v) at room temperature (30 ± 2°C) for 7 days with occasional shaking. The extract was filtered, evaporated, freeze-dried, and stored at −20°C until used for the analysis.

### 2.4. HMG-CoA Reductase Inhibition Assay

The HMG-CoA reductase assay was performed using the HMG-CoA reductase assay kit from Sigma-Aldrich, USA (CS 1090). HMG-CoA (substrate), NADPH (dihydronicotinamide-adenine dinucleotide phosphate), assay buffer, catalytic domain of purified human recombinant enzyme [3-hydroxy-3-methylglutaryl-CoA reductase (HMGR)], and positive control (pravastatin) were supplied with the assay kit. The concentration of the enzyme (HMGR) stock solution was 0.50–0.70 mg/mL. The assay was carried out according to the defined conditions given by the supplier. A reaction volume of 200 *μ*L containing 182 *μ*L of 1x assay buffer, 4 *μ*L of NADPH, and 12 *μ*L of HMG-CoA substrate were initiated by the addition of 2 *μ*L of the catalytic domain of human recombinant HMG-CoA reductase and were incubated at 37°C in the absence (control) or presence of different concentrations of bark extracts (assay concentration -100, 150, and 200 *μ*g/mL) in dimethyl sulfoxide (DMSO). The rates of NADPH consumed were monitored spectrophotometrically in terms of decrease in absorbance at 340 nm using 96-well microplate reader (SpectraMax Plus 384, Molecular Devices, Inc., USA) for 10 minutes with the time interval of 20 sec. The kinetic parameter *V*max was used to calculate the % inhibition compared to control. Pravastatin (assay concentration 0.02–1.25 *μ*g/mL, *n* = 3) was used as the positive control.

### 2.5. Lipase Inhibition Assay

Pancreatic lipase inhibitory activity of bark extracts of Ceylon cinnamon was carried out according to the method described by Kim et al. [22] with some modifications. Reaction volume of 200 *μ*L, containing 30 *μ*L of 2.5 mg/mL porcine pancreatic lipase (PPL, type II) enzyme and 120 *μ*L of different concentrations of bark extracts (assay concentrations: 600, 300, 150, 75, and 37.5 *μ*g/mL) in 0.1 M Tris HCl buffer with 5 mM CaCl_2_, pH 7.0, were preincubated at 37°C for 15 min. Reaction was started by adding 5 *μ*L of 10 mM p-NPB in dimethylformamide and was allowed to proceed at 37°C for 30 min. Lipase inhibitory activity of bark extracts was determined by measuring the hydrolysis of p-NPB to p-nitrophenol at 405 nm using microplate reader. Inhibition of lipase activity was expressed as the percentage decrease in optical density when pancreatic lipase was incubated with bark extracts. Lipase inhibition (%) was calculated according to the following formula and antilipase activity is given as IC_50_ values (the concentrations of bark extracts and the positive control that inhibited the hydrolysis of p-NPB to p-nitrophenol by 50%, *n* = 3). Orlistat was used as the positive control (assay concentration: 0.20–6.25 *μ*g/mL, *n* = 3).

The percentage inhibition was calculated as

(1) $$\begin{array}{c} \text{Inhibition}\;\left(\;\%\;\right)=\frac{\left(A-a\right)-\left(B-b\right)}{\left(A-a\right)}, \end{array}$$

where *A* is the activity without inhibitor, *a* is the negative control without inhibitor, *B* is the activity with inhibitor, and *b* is the negative control with inhibitor.

### 2.6. Cholesterol Esterase Inhibition Assay

Pancreatic cholesterol esterase inhibitory activity of bark extracts of Ceylon cinnamon was performed according to the method reported by Pietsch and Gütschow [21] with minor modifications. Reaction volume of 200 *μ*L, containing different concentrations of bark extracts (assay concentrations: 100, 50, 25, 12.5, 6.25, and 3.125 *μ*g/mL), was preincubated with 50 *μ*L of 24 mM taurocholic acid, 5 *μ*L of 8 mM p-NPB in acetonitrile in 0.1 M sodium phosphate buffer, 0.1 M NaCl, pH 7.0 at 25°C for 10 min. Reaction was started by adding 42.5 *μ*L of (1.25 *μ*g/mL) cholesterol esterase enzyme and change in absorbance was monitored at 405 nm at 25°C for 6 min using SpectraMax 384 microplate reader. The kinetic parameter *V*max was used to calculate the % inhibition and cholesterol esterase inhibitory activity is given as IC_50_ values (the concentrations of bark extracts that inhibited the hydrolysis of p-NPB to p-nitrophenol by 50%, *n* = 4). Simvastatin was used as the positive control (assay concentration: 2.5–30 *μ*g/mL, *n* = 3).

The percentage inhibition was calculated as

(2) $$\begin{array}{c} \text{Inhibition}\;\left(\;\%\;\right)=\frac{\left(A_{C}-A_{S}\right)}{A_{C}}\times 100, \end{array}$$

where *A*_*C*_ is the *V*max of the control and *A*_*S*_ is the *V*max of the sample.

### 2.7. Cholesterol Micellization Inhibition Assay

Artificial micelles were prepared according to the method described by Kirana et al. [20] with minor modifications. Briefly, the solution containing 2 mM cholesterol, 1 mM oleic acid, and 2.4 mM phosphatidylcholine was dissolved in methanol and dried under nitrogen before adding 15 mM phosphate-buffered saline (PBS) containing 6.6 mM taurocholate salt, pH 7.4. The suspension was sonicated twice for 30 min using a sonicator (Bandelin SONOREX Electronic, RK 510) and was incubated at 37°C overnight. Different concentrations of bark extracts (assay concentrations: 250, 500, and 1000 *μ*g/mL; *n* = 6) and PBS as the control were added to the mixed micelle solution and were incubated at 37°C for further 2 h. The solution was centrifuged at 16,000 rpm for 20 min. The supernatant was collected and cholesterol concentration was determined using total cholesterol test kit (BXC0261, Fortress Diagnostics, UK) and cholesterol micellization inhibition is given as IC_50_ values (the concentrations of bark extracts and the positive control that inhibited the solubility of cholesterol by 50%). EGCG was used as the positive control (assay concentration: 250, 500, and 1000 *μ*g/mL, *n* = 3).

### 2.8. Bile Acid Binding Assay

Effects of Ceylon cinnamon bark extracts on bile acid binding were performed according to the method reported by Adisakwattana et al. [9] with some modifications. Taurocholic acid, glycodeoxycholic acid, and chenodeoxycholic acid were used as the bile acids. Briefly, bark extracts (assay concentrations: 3, 2 and 1 mg/mL; *n* = 4) were incubated with each bile acid (2 mM) in 0.1 M phosphate buffer (PBS), pH = 7, at 37°C for 90 min. Each bile acid without extract was used as the control. The mixtures were filtered through 0.22 *μ*m filter to separate the bound bile acids from the free bile acids and were frozen at −20°C until the analysis was carried out. The bile acid concentration was analyzed spectrophotometrically at 540 nm by using bile acid analysis kit (BQ 042A-EALD). Cholestyramine resin was used as the positive control (assay concentration: 3, 2, and 1 mg/mL, *n* = 4).

### 2.9. Quantification of Individual Compounds via HPLC-DAD

Quantification of individual compounds was performed using a Shimadzu HPLC system (Shimadzu, Kyoto, Japan) equipped with LC-10ADVP pump and a SPD-M10AVP diode array detector. Reverse phase chromatographic analysis was carried out with an analytical Phenomenex (Torrance, CA, USA) C 18 Kinetex® 5 *μ*m Phenyl-Hexyl 100 Å pore size (length 250 mm, internal diameter 4.6 mm) column fitted with a guard column. The diode array detector was set at an acquisition range of 200–600 nm. The mobile phase consisted of 2% (v/v) acetic acid in water (eluent A) and methanol and acetonitrile (4.5/4.0, v/v; eluent B). The flow rate was 1 mL/min, and the gradient programme was optimized as follows: 20–37% B (5 min), 37–55% B (7 min), 55–63% B (8 min), 63–37% B (1 min), and 37% B (8 min). Ethanol and DCM : M bark extracts and all the reference standards were filtered through 0.45 *μ*m membrane filter (Millipore) and were degassed by ultrasonic bath prior to injection. Total run time was 35 min and injection volume for all samples and standards were 20 *μ*L. The chromatography peaks were confirmed by comparing their retention time with those of reference standards and by DAD spectra (200–600 nm).

### 2.10. Statistical Analysis

Data of each experiment were statistically analyzed using SAS version 6.12. One-way analysis of variance (ANOVA) and Duncan's Multiple Range Test (DMRT) were used to determine the differences among treatment means. *p* < 0.05 was regarded as significant.

## 3. Results

### 3.1. HMG-CoA Reductase Inhibitory Activity

**Figure 1:**
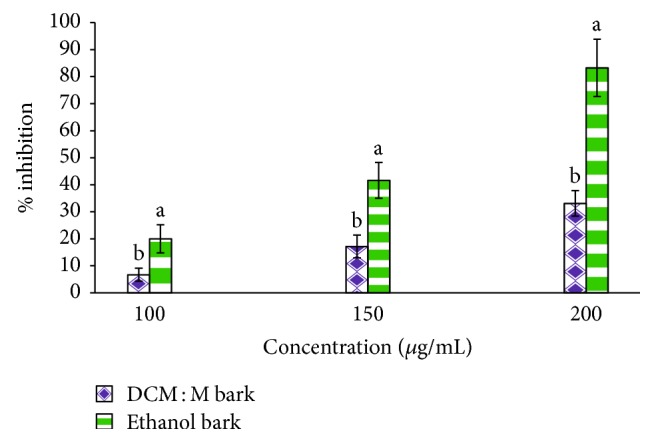
HMG-CoA reductase inhibitory activity of ethanol and DCM : M bark extracts of Ceylon cinnamon. Data represented as mean ± SEM (*n* = 3); mean % inhibition values of ethanol and DCM : M bark extracts superscripted by different letters at each concentration were significantly different at *p* < 0.05. Mean IC_50_ values of ethanol bark, DCM : M, and pravastatin were 153.07 ± 8.38, 277.13 ± 32.18, and 0.50 ± 0.05 *μ*g/mL, respectively. Ethanol bark, DCM : M bark, and pravastatin *r*^2^ = 0.97, 0.99, and 0.99, respectively. DCM : M: dichloromethane : methanol.

HMG-CoA reductase inhibitory activity of ethanol and DCM : M bark extracts of Ceylon cinnamon is given in Figure 1. Both bark extracts demonstrated dose-dependent (ethanol bark *r*^2^ = 0.99 and DCM : M bark *r*^2^ = 0.97) HMG-CoA reductase inhibitory activity. However, ethanol bark extract showed significantly (*p* < 0.05) high activity (IC_50_  153.07 ± 8.38 *μ*g/mL) compared to the DCM : M bark extract (IC_50_  277.13 ± 32.18 *μ*g/mL). Further, both bark extracts showed moderate HMG-CoA reductase inhibition compared to the standard drug pravastatin (IC_50_  0.50 ± 0.05 *μ*g/mL).

### 3.2. Antilipase Activity

Both ethanol and DCM : M bark extracts of Ceylon cinnamon showed moderate and dose-dependent antilipase activity (ethanol bark and DCM : M bark IC_50_: 301.09 ± 4.05 and 297.57 ± 11.78 *μ*g/mL, resp.; *r*^2^ = 0.99 and 0.98, resp.). Further, the antilipase activities of the two extracts were almost similar (*p* < 0.05). Compared to the standard drug orlistat (IC_50_  26.78 ± 2.45 *μ*g/mL), both extracts showed moderate activity. The dose-response relationship of ethanol and DCM : M bark extracts for antilipase activity is given in Table 1.

### 3.3. Anticholesterol Esterase Activity

Cholesterol esterase inhibitory activity of ethanol and DCM : M bark extracts of Ceylon cinnamon is given in Table 2. Both bark extracts possess significant (*p* < 0.05) anticholesterol esterase activity in a dose-dependent manner (ethanol bark *r*^2^ = 0.99 and DCM : M bark *r*^2^ = 0.97). However, ethanol bark extract had significantly high activity compared to DCM : M bark extract (*p* < 0.05). The IC_50_ values of ethanol bark and DCM : M bark extracts were 30.61 ± 0.79 and 34.05 ± 0.41 *μ*g/mL, respectively.

### 3.4. Cholesterol Micellization Inhibitory Activity

Both ethanol and DCM : M bark extracts of Ceylon cinnamon showed cholesterol micellization inhibitory activity in a dose-dependent manner (ethanol bark *r*^2^ = 0.99 and DCM : M bark *r*^2^ = 0.90). However, ethanol bark extract had significantly (*p* < 0.05) high activity compared to DCM : M bark extract. The IC_50_ values of ethanol and DCM : M bark extracts were 231.96 ± 9.22 and 478.89 ± 9.27 *μ*g/mL, respectively. Further, both extracts had moderate cholesterol micellization inhibitory activity compared to standard drug EGCG (150.98 ± 18.72 *μ*g/mL). The dose-response relationship of ethanol and DCM : M bark extracts and EGCG for cholesterol micellization inhibitory activity is given in Table 3.

### 3.5. Binding of Bile Acids by Bark Extracts of Ceylon Cinnamon

**Figure 2:**
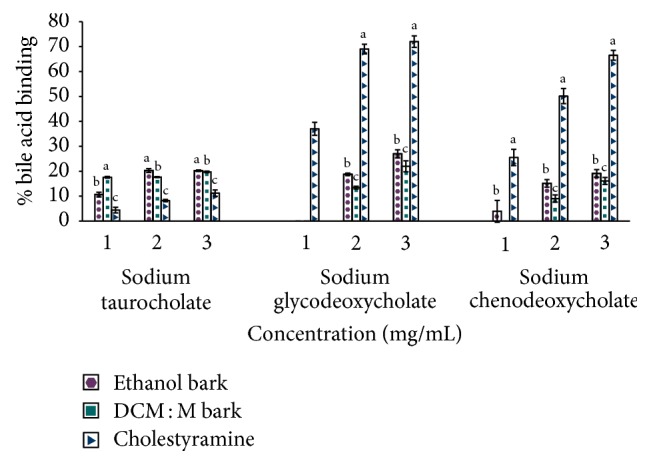
Binding of bile acids by bark extracts of Ceylon cinnamon and cholestyramine. Data represented as mean ± SEM (*n* = 4); statistical analysis was carried out separately for each concentration of each bile acid. Mean % binding values superscripted by different letters at each concentration of each bile acid are significantly different at *p* < 0.05; ethanol bark and DCM : M bark zero binding for sodium glycodeoxycholate at 1 mg/mL; DCM : M bark zero binding for sodium chenodeoxycholate at 1 mg/mL; DCM : M: dichloromethane : methanol.

Effects of ethanol and DCM : M bark extracts of Ceylon cinnamon on binding of three bile acids, namely, sodium taurocholate hydrate, sodium glycodeoxycholate, and sodium chenodeoxycholate are given in Figure 2. Both bark extracts had bile acid binding for the studied three bile acids. However, binding of sodium taurocholate was significantly high (*p* < 0.05) compared to sodium glycodeoxycholate and sodium chenodeoxycholate by both bark extracts. Compared to the standard drug cholestyramine, both bark extracts showed significantly high (*p* < 0.05) sodium taurocholate binding at the studied concentrations of 1, 2, and 3 mg/mL. The percentage binding of sodium taurocholate by ethanol and DCM : M bark extracts and cholestyramine was in the range of 10.66 ± 0.93–20.22 ± 0.3, 17.59 ± 0.31–19.74 ± 0.31, and 4.43 ± 1.15–11.25 ± 1.27, respectively. In contrast, cholestyramine showed significantly high (*p* < 0.05) sodium glycodeoxycholate and sodium chenodeoxycholate binding compared to both bark extracts at the studied concentrations of 1, 2, and 3 mg/mL. The percentage binding of cholestyramine to sodium glycodeoxycholate and sodium chenodeoxycholate was in the range of 37.02 ± 2.59–71.99 ± 2.28 and 25.53 ± 3.30–66.51 ± 1.95, respectively. Further, ethanolic bark extract showed significantly high (*p* < 0.05) sodium glycodeoxycholate and sodium chenodeoxycholate binding compared to DCM : M bark extract. At 3 mg/mL concentration ethanolic bark extract had 26.97 ± 1.61% binding of sodium glycodeoxycholate and 19.11 ± 1.52% binding of sodium chenodeoxycholate, whereas DCM : M bark extract showed 21.97 ± 2.21% binding of sodium glycodeoxycholate and 16.11 ± 1.42% binding of sodium chenodeoxycholate.

### 3.6. Quantification of Ten Individual Compounds in Ethanol and DCM : M Bark Extracts of Ceylon Cinnamon via HPLC-DAD

**Figure 3:**
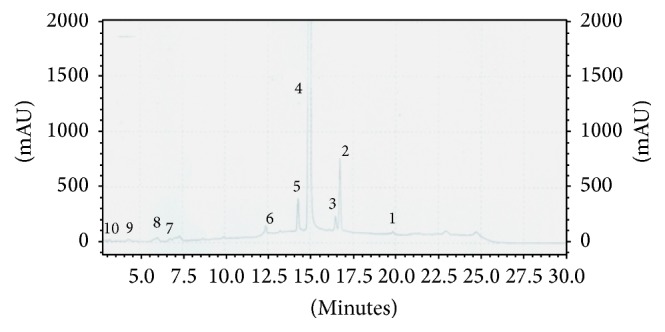
HPLC chromatogram of ethanol bark extract of Ceylon cinnamon. 1: cinnamyl acetate; 2: eugenol; 3: kaempferol; 4: cinnamaldehyde; 5: trans-cinnamic acid; 6: phlorizidin; 7: epicatechin; 8: 4-hydroxybenzoic acid; 9: catechin; 10: gallic acid.

**Figure 4:**
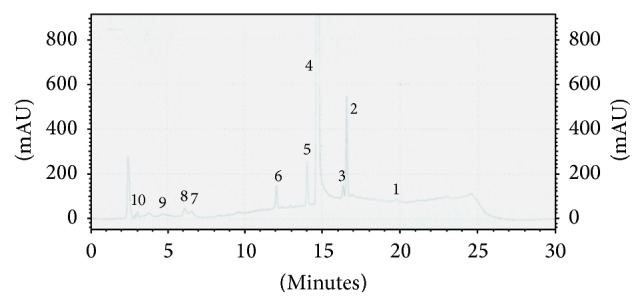
HPLC chromatogram of DCM : M bark extract of Ceylon cinnamon. 1: cinnamyl acetate; 2: eugenol; 3: kaempferol; 4: cinnamaldehyde; 5: trans-cinnamic acid; 6: phlorizidin; 7: epicatechin; 8: 4-hydroxybenzoic acid; 9: catechin; 10: gallic acid.

Both ethanol and DCM : M bark extracts of Ceylon cinnamon had all the tested individual compounds in varying quantities. The quantity of individual compounds in ethanol and DCM : M bark extracts ranged 2.14 ± 0.28–101.91 ± 3.61 and 0.42 ± 0.03–49.12 ± 1.89 mg/g of extract, respectively. Cinnamaldehyde was the highest and gallic acid was the lowest quantified compound in both bark extracts studied. Among the ten tested individual compounds, six individual compounds were present in high quantities in ethanol bark extract (cinnamyl acetate, cinnamaldehyde, epicatechin, catechin, 4-hydroxybenzoic acid, and gallic acid: 11.26 ± 0.17, 101.91 ± 3.61, 10.73 ± 0.73, 17.28 ± 1.65, 11.18 ± 0.23, and 2.14 ± 0.28 mg/g of extract, resp.) compared to DCM : M bark (cinnamyl acetate, cinnamaldehyde, epicatechin, catechin, 4-hydroxybenzoic acid, and gallic acid: 8.92 ± 0.71, 49.12 ± 1.89, 6.90 ± 1.03, 2.90 ± 0.57, 3.26 ± 0.47, and 0.42 ± 0.03 mg/g of extract, resp.). The DCM : M bark extract had high eugenol content (19.98 ± 1.56 mg/g of extract) compared to ethanol bark (13.89 ± 0.14 mg/g of extract). Kaempferol, trans-cinnamic acid, and phlorizidin contents in ethanol and DCM : M bark extracts were statistically insignificant (ethanol and DCM : M bark extracts, kaempferol: 7.04 ± 1.73 and 5.84 ± 1.64 mg/g of extract, resp.; trans-cinnamic acid: 3.33 ± 0.65 and 2.53 ± 0.13 mg/g of extract, resp.; phlorizidin: 4.01 ± 0.36 and 4.47 ± 1.39 mg/g of extract, resp.) (*p* < 0.05). Results of ten individual compounds in both ethanol and DCM : M bark extracts of Ceylon cinnamon are given in Table 4. HPLC chromatograms of both ethanol and DCM : M bark extracts of Ceylon cinnamon are given in Figures 3 and 4 separately.

## 4. Discussion

This study was initiated with a view to develop novel hypolipidemic nutraceuticals and functional foods using bark of true cinnamon for the prevention and management of hyperlipidemia, which is an emerging health problem worldwide [1]. In this connection, the study investigated the antihyperlipidemic potential (mediated via impairment of lipid synthesis, digestion, and absorption) of alba grade bark of true cinnamon by assessing its inhibitory activities on HMG-CoA reductase, lipase, cholesterol esterase, and cholesterol micellization and effect on bile acid binding in vitro. Alba grade cinnamon bark samples were used since it is the most highly priced cinnamon grade in the international trade due to its finest quill thickness, unique aroma, and taste. Ethanol and DCM : M bark extracts were used as those extracts have been previously used in investigation of antioxidant activity [24].

The enzyme 3-hydroxy-3-methylglutaryl-coenzyme A (HMG-CoA) reductase is the rate-limiting enzyme in cholesterol and other isoprenoids biosynthesis that catalyzes the conversion of HMG-CoA to mevalonate [25]. The inhibition of HMG-CoA reductase effectively lowers the level of cholesterol in humans by the activation of sterol regulatory element-binding protein-2, which upregulates the HMG-CoA reductase and LDL receptors that lead to the reduction of cholesterol levels [25], since there are many drugs at clinical level that lower cholesterol level targeted on the HMG-CoA reductase enzyme inhibition [26, 27]. Thus, potent inhibitors of this enzyme can play a key role in the management of hyperlipidemia worldwide [26–29]. The present study showed that both bark extracts of true cinnamon inhibited the HMG-CoA reductase enzyme in a dose-dependent manner (ethanol and DCM : M bark extracts IC_50_: 153.07 ± 8.38  and 277.13 ± 32.18 *μ*g/mL, resp.). Indeed, an investigation carried out by Lee et al. [17] on cinnamate supplementation in high cholesterol-fed rats showed that cinnamate, a phenolic compound found in cinnamon bark, lowers cholesterol levels by inhibiting the HMG-CoA reductase activity. Both ethanol (3.33 ± 0.65 mg/g of extract) and DCM : M (2.53 ± 0.13 mg/g of extract) bark extracts had cinnamate (trans-cinnamic acid) in varying quantities. This may be one possible compound that inhibited the HMG-CoA reductase activity in both ethanol and DCM : M bark extracts of true cinnamon. A recent study carried out by Lopes et al. [30] showed that bark extract of* Cinnamomum zeylanicum* attenuates lipogenic processes via regulating the expression of key enzymes including HMG-CoA reductase enzyme, transcriptional factors, and their target genes, which are directly involved in lipogenesis. Further, HPLC analysis showed cinnamaldehyde as the major phytoconstituent in cinnamon extract. In addition, phenylpropanoids, fatty acids, and procyanidins were also identified [30]. Similarly, in the present study, cinnamaldehyde is the major phytoconstituent in both bark extracts studied (ethanol and DCM : M bark extracts: 101.91 ± 3.61 and 49.12 ± 1.89 mg/g of extract, resp.). Results of total proanthocyanidins also showed that bark extracts contain high contents of total proanthocyanidins [31]. Further, an investigation carried out by Ademosun et al. [32] showed that phenolics from grape fruit peel can inhibit HMG-CoA reductase activity. Among the studied bark extracts, ethanol bark had high activity compared to DCM : M bark may be due to high phenolic content in ethanol bark compared to DCM : M bark [24]. This is the first report on HMG-CoA reductase inhibitory activity of authenticated bark of Ceylon cinnamon worldwide since Lopes et al. [30] carried out the research without proper authentication of the experimental sample.

Recent research findings clearly revealed that fat digestion and absorption as a possible preventive and curative treatment for hyperlipidemia as well as obesity act through gastrointestinal mechanisms [3, 10]. Main stages of fat digestion and absorption include hydrolysis, emulsification, and micelle formation [33]. Intestinal fat digestion is mainly due to the action of pancreatic lipase, a key enzyme involved in the digestion of fats into free fatty acids and glycerol [34]. In fact, therefore, pancreatic lipase inhibition is one of the most widely studied mechanisms for determining natural products potential efficacy as antihyperlipidemic [3, 10] and antiobesity agents [34]. In this study, both bark extracts of true cinnamon showed antilipase activity, which was moderate compared to the clinically available drug orlistat, a derivative of the naturally occurring lipase inhibitor produced from* Streptomyces toxytricini* [35]. It is the only approved lipase inhibitor at clinical level to treat obesity to date. However, unpleasant gastrointestinal side effects including oily spotting, liquid stools, fecal urgency or incontinence, flatulence, and abdominal cramping necessitate the search of novel inhibitors, derived from plants or other natural sources that lack some of these unpleasant side effects [34]. Several polyphenolics such as flavones, flavonols, tannins, procyanidins, and chalcones are reported to have lipase inhibition [34]. In the present study, 10 individual compounds were quantified in both bark extracts of true cinnamon and some of the quantified compounds such as gallic acid, kaempferol, catechin, and epicatechin reported to have lipase inhibition [36]. In our previous studies, Ceylon cinnamon bark extracts were also shown to have high phenolic contents [24] including proanthocyanidins [31]. Further, both bark extracts showed antioxidant properties via multiple mechanisms [24]. Research carried out by Ngamukote et al. [37] reported that the effect of grape seed extract on lipase inhibition might be caused by a synergistic action of several compounds within the extract, namely, flavonoids, procyanidins, and their antioxidative metabolites, rather than by a single compound. Therefore, the observed antilipase activity of bark extracts of Ceylon cinnamon may be attributed, at least partly, to phenolic compounds including proanthocyanidins and other antioxidants present in both bark extracts [24, 36, 37]. In fact,* Cinnamomum* species are scarcely investigated for antilipase activity worldwide. Gholamhoseinian et al. [38] reported that methanolic extract of derm of* Cinnamomum zeylanicum* possesses 39% inhibition of lipase activity at 50 *μ*g/mL concentration using turbidimetric method. A recent study conducted by McCrea et al. [39] reported that acetone : water : acetic acid (80 : 20 : 1) extract of bark of cinnamon had potent antilipase activity as IC_50_: 5.5 *μ*g/mL. However, the cinnamon species used in the study is not mentioned. Those two investigations are the only available previous reports on antilipase activity of bark of cinnamon and results showed significant variation compared to the present study. Therefore, the discrepancy observed between present study and previous investigations on antilipase activity may be due to the use of different assay methods, extraction procedures, and use of cinnamon samples without proper authentication.

Dietary fat absorption by the small intestine is a multistep process and its impairment is mediated by inhibition of pancreatic cholesterol esterase [8], cholesterol micellization [7, 40], and bile acid binding [10]. Dietary cholesterol consists of both free and esterified cholesterols and esterified cholesterols are hydrolysed by pancreatic cholesterol esterase [8]. Moreover, it plays an important role in regulating the incorporation of cholesterol into mixed micelles [41]. Therefore, inhibition of cholesterol esterase activity plays an important role in reduction of dietary cholesterol absorption. The next principal steps involved in absorption of dietary cholesterol are micellar solubilization and bile acid binding. Moreover, bind bile acids form insoluble complexes and thereby increase fecal excretion and lower plasma cholesterol level [42]. Therefore, reduction of cholesterol absorption by inhibition of micellar solubilization and binding of bile acid is now considered as a new target site of intervention for treatment of hyperlipidemia and obesity worldwide [42, 43]. Some phenolic compounds such as gallic acid, catechin, and epicatechin showed inhibitory activity against cholesterol esterase and cholesterol micellization and also facilitate bile acid binding in vitro [37]. Further, it is also reported that flavonoids act as suicide substrate ahead of cholesterol esters [8]. The results of the present study showed that both bark extracts of true cinnamon possess moderate cholesterol esterase and cholesterol micellization inhibition and bile acid binding in vitro. Since phenolics were shown to have inhibitory activities on cholesterol esterase and cholesterol micellization and bile acid binding, it can be hypothesized that these activities may be due the rich phenolic content in both bark extracts [24]. Interestingly, this is also the first report on cholesterol esterase and cholesterol micellization inhibition and bile acid binding by bark of Ceylon cinnamon in vitro.

We have previously reported marked antioxidant properties of bark extracts of Ceylon cinnamon mediated via multiple mechanisms [24]. Oxidative stress is now known to be involved in hyperlipidemia; it is indeed an early event in the development of hyperlipidemia [44]. As free radicals are involved in lipid peroxidation and related hyperlipidemic activities, antioxidants can play a vital role in antilipidemic activities. Therefore, observed antilipidemic activities of bark extracts of Ceylon cinnamon may be at least partly due to the presence of antioxidative compounds. Further, experiments are in progress to isolate active compounds and efficacy in vivo studies.

Several recent research studies have shown that bark of* C. zeylanicum *possesses lipid-lowering activity in vivo. The research carried out by Hassan et al. [18] reported that aqueous bark extract of* C. zeylanicum* reduced total and LDL cholesterol and triglycerides while increasing HDL-cholesterol in streptozotocin-induced type 1 diabetes mellitus (T1DM) rats. Another study carried out by Ranasinghe et al. [10] confirmed that bark of* C. zeylanicum* significantly lowers the total and LDL cholesterol levels on day 30 compared to day 0 in both diabetic and healthy rats. Further, Javed et al. [19] have shown that lipid-lowering effect of methanolic extract of bark of* C. zeylanicum* at 0.75 g/kg of body weight was similar to the lipid-lowering effect of simvastatin at 0.6 mg/kg body weight in hyperlipidemic albino rabbits. However, research studies conducted so far on antilipidemic activity of bark of* C. zeylanicum* do not clearly described the mechanisms of lipid lowering [10]. It is possible that the lipid-lowering activity observed in in vivo investigations [10, 18, 19] may be due to one or more activities evident in the present study. Moreover, to date, there are no clinical level investigations on antilipidemic activity of bark of Ceylon cinnamon. The present study confirmed the antilipidemic potential of bark of authenticated Ceylon cinnamon mediated via HMG-CoA reductase, lipase, cholesterol esterase, and cholesterol micellization inhibitory activities and bile acids binding in vitro. Interestingly, studied antilipidemic activities of bark of Ceylon cinnamon including HMG-CoA reductase, cholesterol esterase, and cholesterol micellization inhibitory activities and bile acids binding are novel findings. We hope that important findings on antilipidemic properties of Ceylon cinnamon demonstrated here would help to enhance its consumption among consumers locally and internationally and it may create a positive financial impact to Sri Lanka as, currently, Ceylon cinnamon is the true cinnamon the world over and the main contributor of the export earnings from spices in the country.

## 5. Conclusions

It is concluded that bark of Ceylon cinnamon “true cinnamon” possesses HMG-CoA reductase, lipase, cholesterol esterase, and cholesterol micellization inhibitory activities and bind bile acids in vitro. Thus, consumption of Ceylon cinnamon bark as a dietary supplement may play a vital role in the management of hyperlipidemia and obesity worldwide. Most importantly, the findings of this study add value to bark of Ceylon cinnamon and indicate its huge potential in developing promising novel hypolipidemic food supplements, nutraceuticals, functional foods, and use in adjuvant therapy in the management of hyperlipidemia and obesity worldwide.

## Figures and Tables

**Table 1:** Antilipase activity of bark extracts of Ceylon cinnamon and orlistat.

Extract/Std	% inhibition					*µ*g/mL
	Concentration (*µ*g/mL)					
Bark	37.5	75	150	300	600	IC_50_

Ethanol	5.78 ± 0.57	6.77 ± 0.90	24.50 ± 3.13	49.89 ± 0.21	55.87 ± 2.54	301.09 ± 4.05^a^
DCM : M	12.50 ± 0.93	17.95 ± 4.04	24.14 ± 2.20	52.07 ± 1.39	55.66 ± 2.17	297.57 ± 11.78^a^

Std	0.01	0.39	1.56	6.25	25	IC_50_

Orlistat	18.85 ± 0.31	27.84 ± 0.61	34.62 ± 0.67	41.54 ± 0.55	49.69 ± 0.65	26.78 ± 2.45^b^

Data represented as mean ± SEM (*n* = 3); mean IC_50_ values superscripted by different letters are significantly different at *p* < 0.05. Ethanol bark, DCM : M bark, and orlistat are *r*^2^ = 0.99, 0.98, and 0.98, respectively. DCM : M: dichloromethane : methanol.

**Table 2:** Cholesterol esterase inhibitory activity of bark extracts of Ceylon cinnamon and simvastatin.

Extract/Std	% inhibition					*µ*g/mL
	Concentration (*µ*g/mL)					
Bark	3.125	6.25	12.5	25	50	IC_50_

Ethanol	1.85 ± 0.58	12.49 ± 1.50	32.55 ± 0.32	48.65 ± 0.61	58.30 ± 1.12	30.61 ± 0.79^b^
DCM : M	2.44 ± 1.24	8.74 ± 0.29	27.47 ± 0.68	46.09 ± 0.58	57.99 ± 0.25	34.05 ± 0.41^a^

Std	2.5	5	10	20	30	IC_50_

Simvastatin	18.49 ± 0.57	27.09 ± 1.16	34.38 ± 1.83	50.00 ± 3.33	73.75 ± 4.31	18.56 ± 0.68^c^

Data represented as mean ± SEM (*n* = 4). Mean IC_50_ values superscripted by different letters are significantly different at *p* < 0.05. Ethanol bark, DCM : M bark, and simvastatin are *r*^2^ = 0.99, 0.98, and 0.99, respectively. DCM : M: dichloromethane : methanol.

**Table 3:** Cholesterol micellization inhibitory activity of bark extracts and EGCG.

Extract/Std	% inhibition of cholesterol solubility in micelles			*µ*g/mL IC_50_
	Concentration (*µ*g/mL)			
	250	500	1000	
EGCG	55.16 ± 0.91	69.78 ± 1.82	96.75 ± 1.70	150.98 ± 18.72^e^
Ethanol bark	49.48 ± 0.78	69.48 ± 0.81	98.09 ± 0.51	231.96 ± 9.22^d^
DCM : M bark	19.36 ± 2.04	62.15 ± 1.06	73.94 ± 0.87	478.89 ± 9.27^c^

Data represented as mean ± SEM (EGCG *n* = 3; ethanol bark and DCM : M bark *n* = 6 each). IC_50_ values in a column superscripted by different letters are significantly different at *p* < 0.05. EGCG, ethanol bark, and DCM : M bark are *r*^2^ = 1.00, 0.99, and 0.90, respectively. DCM : M: dichloromethane : methanol; EGCG: epigallocatechin gallate.

**Table 4:** Quantification of individual compounds in bark extracts of Ceylon cinnamon.

Compound	(mg/g of extract)	
	Ethanol bark	DCM : M Bark
Cinnamyl acetate	11.26 ± 0.17^a^	8.92 ± 0.71^b^
Eugenol	13.89 ± 0.14^b^	19.98 ± 1.56^a^
Kaempferol	7.04 ± 1.73^a^	5.84 ± 1.64^a^
Cinnamaldehyde	101.91 ± 3.61^a^	49.12 ± 1.89^b^
trans-Cinnamic acid	3.33 ± 0.65^a^	2.53 ± 0.13^a^
Phlorizidin	4.01 ± 0.36^a^	4.47 ± 1.39^a^
Epicatechin	10.73 ± 0.73^a^	6.90 ± 1.03^b^
Catechin	17.28 ± 1.65^a^	2.90 ± 0.57^b^
4-Hydroxybenzoic acid	11.18 ± 0.23^a^	3.26 ± 0.47^b^
Gallic acid	2.14 ± 0.28^a^	0.42 ± 0.03^b^

Data represented as mean ± SEM (*n* = 3); bark extracts were separately analyzed for each phenolic compound; mean values in the column superscripted by different letters for each phenolic compound within ethanol and DCM : M bark extracts were significantly different at *p* < 0.05. DCM : M: dichloromethane : methanol.
